# Millimeter-wave sensor based on a λ/2-line resonator for identification and dielectric characterization of non-ionic surfactants

**DOI:** 10.1038/srep19523

**Published:** 2016-01-20

**Authors:** H. Rodilla, A. A. Kim, G. D. M. Jeffries, J. Vukusic, A. Jesorka, J. Stake

**Affiliations:** 1 Department of Microtechnology and Nanoscience, Chalmers University of Technology, SE-41296 Göteborg, Sweden; 2 Department of Chemistry and Chemical Engineering, Chalmers University of Technology, SE-41296 Göteborg, Sweden; 3 Department of Physiology and Pharmacology, Karolinska Institutet, SE-17177 Stockholm, Sweden

## Abstract

Studies of biological and artificial membrane systems, such as niosomes, currently rely on the use of fluorescent tags, which can influence the system under investigation. For this reason, the development of label-free, non-invasive detection techniques is of great interest. We demonstrate an open-volume label-free millimeter-wave sensing platform based on a coplanar waveguide, developed for identification and characterization of niosome constituents. A design based on a λ/2-line resonator was used and on-wafer measurements of transmission and reflection parameters were performed up to 110 GHz. Our sensor was able to clearly distinguish between common niosome constituents, non-ionic surfactants Tween 20 and Span 80, measuring a resonance shift of 3 GHz between them. The complex permittivities of the molecular compounds have been extracted. Our results indicate insignificant frequency dependence in the investigated frequency range (3 GHz – 110 GHz). Values of permittivity around 3.0 + 0.7i and 2.2 + 0.4i were obtained for Tween 20 and Span 80, respectively.

The study and development of artificially formed vesicles has significant relevance due to their role as carriers for drug delivery applications^1^, and they also play key importance in the fields of nutrients delivery^2^ and cosmetics^3^. The prepared vesicles are self-assembled structures formed from amphiphilic molecules, commonly lipids. However, vesicles based on non-ionic surfactants, known as niosomes, have become increasingly popular in pharmaceutical research, due to their chemical stability, shelf-life and lower costs^4^, but most notably for their ability to deliver compounds across the blood-brain barrier^5^. Current detection and analysis schemes, for niosomes and other vesicles, rely heavily on the use of fluorescent tags, which may influence the measurements or perturb the system under investigation. To this end, we investigated the possibility of using dielectric impedance spectroscopy as an alternative detection scheme, as it is a non-invasive, label-free technique which can probe a sample in its native environment without the need for chemical modifications. In addition, dielectric impedance spectroscopy in the millimeter-wave (30–300 GHz) frequency region has interesting implications in chemistry and life science research, as it contains information about molecular rotations, dielectric relaxation and hydrogen bond interactions^6^.

Most of the previous work in millimeter-wave (mm-wave) technology for life science applications extends up to 40 GHz and utilizes microfluidic confinement^7^,^8^,^9^,^10^. Measurements up to 110 GHz in an open-volume arrangement have been reported on yeast cells suspensions, based on a straight coplanar waveguide (CPW) line^11^. However, due to the structural similarity between non-ionic surfactants, in order to increase device selectivity, we developed a sensor based on a λ/2-line resonator, in contrast to the straight CPW implementations^7^,^8^,^9^,^10^,^11^. The mm-wave sensor was implemented in an open-volume configuration to permit facile sample manipulation, creating a versatile platform for investigation of dielectric properties from various types of samples in a label-free, non-invasive manner. In this paper, the sensor was specifically tailored for non-ionic surfactants and utilized for investigation of their complex dielectric permittivities, as substantial knowledge of the behavior of niosome constituents as well as the role of compositional variances in this frequency range is needed in order to develop a dielectric impedance spectroscopy detection and analysis scheme for niosomes and other biologically relevant systems.

## Results

**Figure 1:**
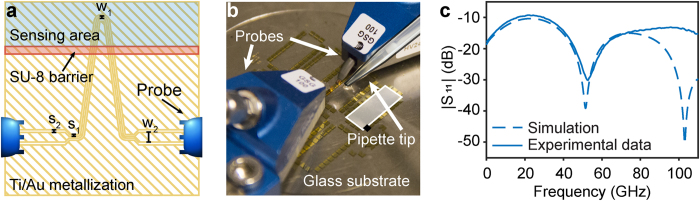
The mm-wave sensor based on a λ/2-line resonator. (**a**) A schematic plot of the fabricated sensor, illustrating the critical length scales w (signal width) and s (signal-to-ground spacer): W_1_, S_1_, W_2_ and S_2_, are 10, 10, 50 and 14 μm, respectively. (**b**) A photograph of the experimental setup. The white box represents a pool formed by the SU-8 barrier and the black box one of the sensors. The tip of the pipette used to transfer fluid to the open volume is also shown. (**c**) Comparison of the 3D electromagnetic simulation (dashed blue line) and the experimental (continuous blue line) magnitude of S_11_ for air in the sensing area.

The mm-wave sensor, based upon a CPW line^12^, was designed to provide high surface sensitivity and enable on-wafer scattering parameters (S-parameters) measurements using 3D electromagnetic simulations. The effective permittivity of a CPW is given by the relationship between the permittivities of the substrate and the material above the sensor^13^. In order to improve the selectivity of the sensor, a λ/2-line resonator was designed. The resonator comprises a 1.3 mm long high impedance line (80 Ω) between two 50 Ω lines (system impedance), exhibiting a selective resonance between 40 and 60 GHz for the compounds investigated in this paper, when the electrical length of the high impedance section is 180 degrees (Fig. 1a). This resonance is sensitive to the effective electrical length of the line, and as a result, it will shift due to the changes in permittivity of the different compounds. The sensor was designed with a bended line configuration, which minimizes liquid-sensor contact, in order to minimize the power losses, while maintaining the open-volume configuration. Figure 1a shows the schematic illustration of the microfabricated sensor, where a confined area for the liquid under investigation is defined by a polymer barrier. In this study, the open-volume arrangement is particular useful as the molecular compounds under investigation are of high viscosity, and this setup allows for easy liquid manipulation (Fig. 1b), as well as cleaning and re-use of the sensor surface.

The experimental results were obtained by on-wafer S-parameter measurements (phase and magnitude of the transmitted (S_21_), and reflected (S_11_) power), from 10 MHz to 110 GHz with the reference plane at the probe tips. In order to minimize the experimentally observed substrate modes (a typical finding at this frequency range), a doped Si wafer was located underneath our glass substrate^14^.

Figure 1c shows the comparison between the experimental results and the 3D electromagnetic simulations for the empty sensor (air) in the magnitude of the S_11_ (reflection) parameter, for the verification of our simulation model. The simulations suggest that a change of ±0.1 in the permittivity of the molecular compound is translated into a resonance frequency shift of ±0.4 GHz, highlighting the potential of our sensor for molecular compound identification.

**Figure 2:**
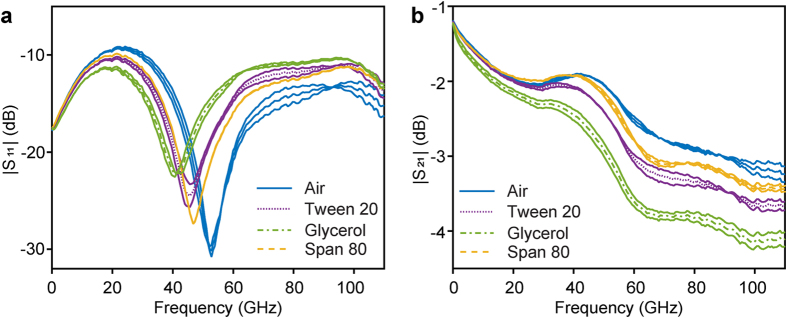
Experimental data of S-parameters versus frequency. Magnitude of (**a**) S_11_ and (**b**) S_21_ for air (blue line), the non-ionic surfactants Tween 20 (dotted purple line) and Span 80 (dashed yellow line) and glycerol (green dash-dotted line). The standard deviation for each compound are included as continuous lines in the plot.

The motivation for the final use of the presented platform is to study niosome membranes, therefore two commonly used non-ionic surfactants for niosome formation, sorbitane monooleate (Span 80) and polyethylene glycol sorbitan monolaurate (Tween 20) were investigated. Moreover, glycerol was included in the study (in contrast to non-ionic surfactants) as it is a small polar molecule, often added in preparations of artificial biological models. In order to verify the repeatability of our results, each compound of interest was measured seven times. Figure 2 presents the mean values of the magnitude of S_11_ (reflection) and S_21_ (transmission) parameters for air, Tween 20, Span 80 and glycerol. The standard deviations of the measurements are included in the plots, demonstrating the consistency of our findings. The differences in the permittivity of the measured compounds invoke not only a change in magnitude of the reflection, but also of the expected shift in the resonance, confirming the high selectivity of our sensor. For the structurally similar compounds Tween 20 and Span 80, a significant resonance shift of 3 GHz was observed. A shift of 6 GHz was observed between Tween 20 and glycerol. An increase of the standard deviation has been observed from around 60 to 80 GHz and around 100 GHz (Fig. 2a), as a consequence of the substrate modes. The implementation of a Si wafer underneath our substrates, reduced the substrate modes. However, this signal component is not completely eliminated, slightly decreasing the stability and therefore increasing the uncertainty of the frequencies, where it is present. If the substrate modes appear within the frequency range of interest, there are methods (such as thinning down the substrate) to displace them to a higher frequency. This was not necessary in our case, as their influence on the standard deviation of the measurements was minimal and occurred outside the resonance area of the compounds investigated.

**Figure 3:**
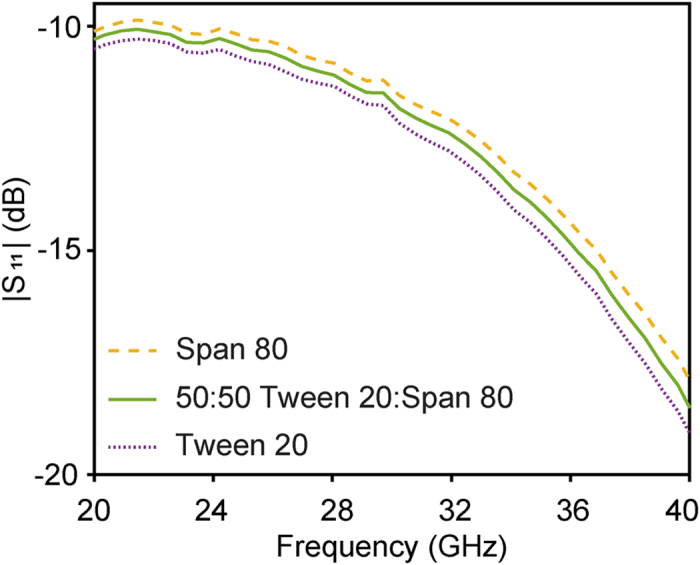
Comparison between the 50% (w/w) Tween 20 and Span 80 mixture and the pure components. Experimental magnitude of S_11_ versus frequency for Tween 20 (dotted purple line), Span 80 (dashed yellow line) and the mixture (solid green line).

Air, Span 80, Tween 20 and glycerol are also distinguishable in the magnitude of S_21_, as seen in Fig. 2b. In order to further investigate the selectivity of our sensor a 50% (w/w) mixture of Tween 20 and Span 80 was compared with pure Tween 20 and Span 80. The results, emphasized for an arbitrary frequency range in Fig. 3, show that the data for the mixture is in between the pure compounds, illustrating the high performance of our sensor.

**Figure 4:**
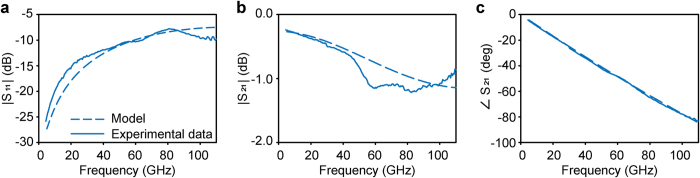
Comparison of the experimental data (continuous blue line) and the circuit model (dashed blue line) for air. Magnitude of (**a**) S_11_ and (**b**) S_21_ and (**c**) phase of S_21_ for the empty sensor with the reference plane after the SU-8 barrier.

In order to obtain the complex dielectric permittivities, first, a de-embedding procedure for the frequency range 3 to 110 GHz was established for moving the reference plane from the probe tips to the sensing area inside the open volume. For this, Thru-Reflect-Line (TRL) calibration^15^ structures to cover the frequency range (3 – 110 GHz) were designed and fabricated. In the second step, we performed circuit modelling simulation of a CPW with the same dimensions and materials as the fabricated sensor, taking the empty sensor (air) as a reference. The experimental results for air were de-embedded and compared with the results from the circuit modelling simulation. Figure 4 shows the agreement between the experimental and modelled results for air. Despite the overall good agreement between the experimental results and the circuit model, the propagation of the substrate modes in the experimental case, though minimized by the use of the Si wafer, still causes some small (less than 0.5 dB, Fig. 4a,b) discrepancies between the experimental and model results (see Supplementary Information S2).

**Figure 5:**
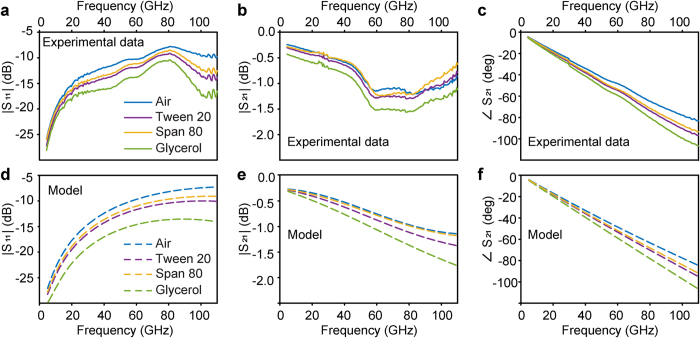
Comparison of the experimental data after the de-embedding process (**a–c**, continuous lines) and the circuit model with adjusted values of the permitivity (**d–f**, dashed lines). The magnitude of S_11_ (**a,d**), S_21_ (**b,e**) and the phase of S_21_ (**c,f**). Air (blue), Tween 20 (purple), Span 80 (yellow) and glycerol (green).

Figure 5a–c presents the experimental data for air, Span 80, Tween 20 and glycerol after the de-embedding procedure (when the reference plane is moved to the sensing area). The compounds investigated in this study show low losses in this frequency range, with glycerol having the highest losses among them. Figure 5c shows a constant slope of the phase of S_21_ versus frequency, indicating an insignificant frequency dependence of the real part of the permittivity for the frequency range investigated (3 GHz to 110 GHz). There are only two negligible changes in the slope of the phase of S_21_ at 30 GHz and 60 GHz, artificially introduced by a small instability in the measurements around 30 GHz and the remaining effect of the substrate modes. Due to previous considerations, a constant permittivity was used in the procedure for extracting the complex permittivities. Permittivity values of the molecular compounds under study have been obtained by modifying the permittivity of the material on top of the sensor in the circuit model (verified with air), to reproduce the experimental results. Figure 5d–f show the model results for values of permittivity of 4.8 + 1.6i, 3.0 + 0.7i, and 2.2 + 0.4i for glycerol, Tween 20, and Span 80, respectively. Greater detail about the permittivity subtraction method can be found in Supplementary Information Section S3. To date, little literature information is available for the frequency range investigated. Our results are in agreement with the permittivity data earlier reported for glycerol^16^,^17^. However, the literature data available for non-ionic surfactants is limited to measurements at lower frequencies^18^.

## Conclusion

In this work we demonstrate an open-volume sensing platform for identification and characterizing non-ionic surfactants in the millimeter-wave frequency range based on a λ/2-line resonator. The sensor was able to clearly distinguish between glycerol, Span 80, Tween 20 and a 50% mixture of Tween 20 and Span 80. On-wafer measurements of S-parameters up to 110 GHz were performed, with the sensor design allowing scaling for even higher frequencies. Calibration structures and modelling results in agreement with agreement with experimental data for the empty sensor (air), allow the extraction of the complex permittivity of the molecular compounds under investigation. The permittivities of Tween 20, Span 80 and glycerol have shown to not have a significant frequency dependence between 3 GHz and 110 GHz. Values around 4.8 + 1.6i, 3.0 + 0.7i and 2.2 + 0.4i have been obtained for glycerol, Tween 20 and Span 80 respectively. The selectivity of our sensor could be exploited for detection and identification of surfactant mixtures.

Advances in drug delivery schemes have demonstrated potential applications of niosomes to treat neuronal disorders. One question remains largely unsolved, regarding the physicokinetics and the chemical interactions between niosomes and the blood-brain-barrier. The development of a label-free millimeter-wave platform aids in the understanding of the behavior of amphiphilic molecules, the first step towards real-time monitoring of niosomes.

## Methods

### Fabrication

The sensors were fabricated using standard lift-off procedure. The Ti/Au metallization that forms the CPWs was evaporated (10/300 nm) onto glass substrates. A barrier of 50 μm height was photolithographically patterned on top of the CPW, employing the negative epoxy photoresist SU-8 (2035 MicroChem Corp., USA). Further details of the fabrication procedure can be found in Supplementary Information S4.

### Measurements

The S-parameter measurements (phase and magnitude of the transmitted (S_21_), and reflected (S_11_) power) were performed using an Anritsu Vector Network Analyzer (VNA), from 10 MHz to 110 GHz. The Line-Reflect-Reflect-Match (LRRM) calibration was used together with ISS calibration structures #104-783 from Cascade Microtech to set the reference plane at the probe tips. A 380 μm thick Si wafer, doped to a conductivity of 10 S/m, was located underneath our glass substrate.

## Additional Information

**How to cite this article**: Rodilla, H. *et al.* Millimeter-wave sensor based on a λ/2-line resonator for identification and dielectric characterization of non-ionic surfactants. *Sci. Rep.* **6**, 19523; doi: 10.1038/srep19523 (2016).

## Supplementary Material

Supplementary Information
