# Supplementary material for: Millimeter-wave sensor based on a λ/2-line resonator for identification and dielectric characterization of non-ionic surfactants
Source: Sci Rep. 2016 Jan 20;6:19523. doi: 10.1038/srep19523 (PMC4726352; doi:10.1038/srep19523)
Supplement: Supplementary Information [file srep19523-s1.pdf]

Supplementary Information

Millimeter-wave sensor based on a λ/2 resonator for identification and dielectric characterization of non-ionic surfactants

H. Rodilla[1,a], A. A. Kim[2,3,a], G. D. M. Jeffries[2], J. Vukusic[1], A. Jesorka[2] and J. Stake[1*]

[1] Department of Microtechnology and Nanoscience, Chalmers University of Technology, SE-41296 Göteborg, Sweden.

[2] Department of Chemistry and Chemical Engineering, Chalmers University of Technology, SE-41296 Göteborg, Sweden.

[3] Department of Physiology and Pharmacology, Karolinska Institutet, SE-17177 Stockholm, Sweden.

[a] These authors contributed equally to this work.

[*]jan.stake@chalmers.se

Contents

S1 – Calibration method 3
S2 – Power loss through the substrate 4
S3 – Permittivity subtraction 5
S4 – Microfabrication 6
Patterning of the sensor 6
Patterning of the SU-8 barrier 7
References 8

S1 – Calibration method

The Line-Reflect-Reflect-Match (LRRM) calibration was used together with ISS calibration structures #104-783 from Cascade Microtech to set the reference plane at the probe tips.

Thru-Reflect-Line (TRL) calibration[1] structures to cover the frequency range from 3 GHz to 110 GHz were designed, fabricated and measured in order to move the reference plane from the probe tips to the sensing area inside the pool. This procedure allowed us to obtain the complex permittivity of the compounds under study. The influence of the coupling consequence of the bending design of our sensors has been studied showing no influence on the results. Due to the lack of coupling observed, and in order to have well designed length on the lines, non-bended structures were used in the calibration structures used in the de-embedding process in this paper.

S2 – Power loss through the substrate

The power loss versus frequency for air for the experimental results with the sensor empty (air) once the de-embedding procedure has been applied (calibration plane has been moved to the sensing area) is shown in Fig. S1. An increase of the power loss is observed around 60 GHz, where the substrate modes were observed before locating the Si wafer underneath our sensor. Even though the substrate modes are significantly minimized by the Si wafer, there are still some remaining effect, which is the reason for the small discrepancies in the comparison between the modelled and the experimental data in Fig. 4.

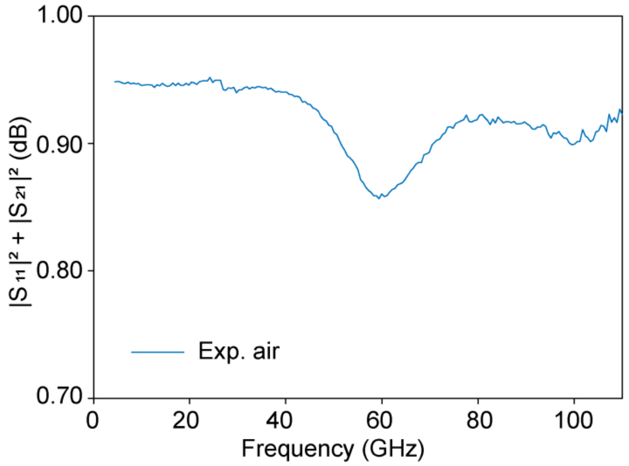

Figure S1. **$|S_{11}|^2 + |S_{21}|^2$ for the experimental measurements of the empty sensor with the reference plane after the SU-8 barrier.**

S3 – Permittivity subtraction

First we performed circuit model simulations of a CPW with the dimensions and materials of the fabricated one and we compared with the experimental results obtained with air in the sensing area. A good agreement between the model and the experimental data was observed (Fig. 4), and this structure was used as reference.

In order extract the permittivity of the compounds under study the circuit model for air as described above was used. The model was modified for each compound by changing the permittivity of the material on top the sensing area to fit the corresponding experimental results. As explained in the paper, we have experimentally observed almost no frequency dependency of the permittivity for the molecular compounds under study. Consequently, a constant permittivity has been used in the model.

In this way, the complex permittivity of the surfactant could be extracted, obtaining values of 4.8+ 1.6i, 3.0 + 0.7i and 2.2 + 0.4i for glycerol, Tween 20 and Span 80 respectively.

S4 – Microfabrication

Patterning of the sensor

The sensors were prepared at the Nanofabrication Laboratory at MC2, Chalmers University of Technology using the following procedure. The schematic illustration of the fabrication procedure can be seen below in Fig. S2.

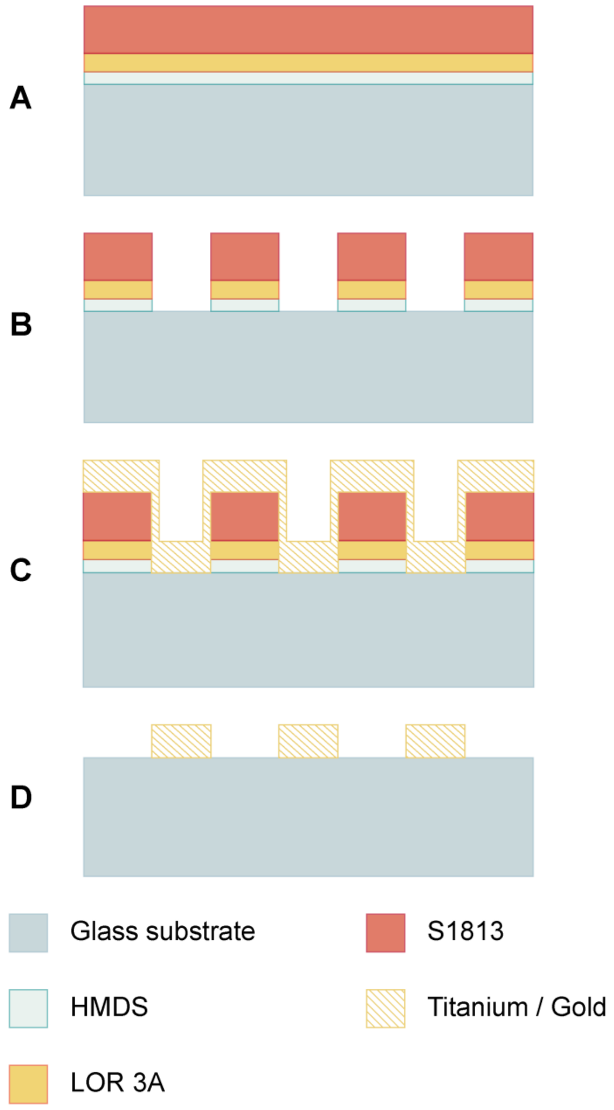

Figure S2. Schematic illustration of the fabrication procedure of the sensors.

Preparation

The glass substrate (Ø 47 mm, thickness #1.5 (0.16-0.19 mm) microscope coverslips, Menzel-Gläser, Germany) was cleaned in a megasonic bath at room temperature for 10 min. The substrate was then rinsed several times and dried using a nitrogen stream. The substrate was treated with oxygen plasma in BatchTop (Plasma Therm, USA) for 30 s, at 50 W, 250 mTorr and 10 sccm O_2.

Patterning

The substrate was dehydrated for 30 seconds at 180^{0}C and directly after HMDS (MicroChemicals GmbH, Germany) was spun at 2000 rpm for 5 s. LOR3A (MicroChem Corp., USA) was deposited and spun at 2000 rpm for 45 s. The substrate was soft-baked at 180^{0}C for 5 min. S1813 (Rohm and Haas Co., USA) was deposited and spun at 3000 rpm for 45 s. The substrate was soft-baked again for 2 min at 110^{0}C (Fig. S1A). The substrate was exposed using mask aligner MA6 (SÜSS MicroTec, Germany) (6 mW/cm^2) for 9 s. The substrate was developed in developer MF-319 (Rohm and Haas Co., USA) for approximately 90 s and checked using optical microscopy. The substrate was rinsed with water and dried using a stream of nitrogen. The substrate was treated with oxygen plasma in BatchTop for 30 s, at 50 W, 250 mTorr and 10 sccm O_2 (Fig. S1B).

Metal deposition and lift-off

Metallization: 10 nm of titanium (Ti) and 300 nm of gold (Au) were deposited using evaporation in Lesker (Kurt J. Lesker Co., USA) onto the substrate (Fig. S1C). The substrate was left overnight in NMP-free remover, mr-Rem 400. The substrate was thoroughly cleaned the following day and treated with oxygen plasma in BatchTop for 30 s, at 50 W, 250 mTorr and 10 sccm O_2 (Fig. S1D).

Patterning of the SU-8 barrier

SU-8 2035 (MicroChem Corp., USA) was deposited and spun, first for 10 s at 500 rpm and then at 3000 rpm for 30 s. The substrate was soft-baked for 12 min at 95^{0}C. The substrate was exposed for 37.5 s in the mask aligner. The substrate was post-exposure baked for 10 min at 95^{0}C. The substrate was developed in mrDev 600, rinsed with isopropyl alcohol and water. The substrate was treated with oxygen plasma in BatchTop for 30 s, at 50 W, 250 mTorr and 10 sccm O_2. The substrate was hard baked in order to fully crosslink SU-8 at 200^{0}C for 30 min with gentle temperature ramping.

Prior to use, the substrate was cleaned with a 0.5% solution of Triton-X (Sigma-Aldrich, Sweden) to remove organic impurities and treated with oxygen plasma in BatchTop for 30 s, at 50 W, 250 mTorr and 10 sccm O_2.

References

1. G. F. Engen and C. A. Hoer, *Microwave Theory and Techniques, IEEE Transactions on*, 1979, **27**, 987-993.
